# Pleiotropic modes of action in tumor cells of RNASET2, an evolutionary highly conserved extracellular RNase

**DOI:** 10.18632/oncotarget.3490

**Published:** 2015-03-08

**Authors:** Marta Lualdi, Edoardo Pedrini, Katia Rea, Laura Monti, Debora Scaldaferri, Marzia Gariboldi, Annalisa Camporeale, Paolo Ghia, Elena Monti, Antonella Tomassetti, Francesco Acquati, Roberto Taramelli

**Affiliations:** ^1^ Department of Theoretical and Applied Sciences, Università degli Studi dell'Insubria, Varese, Italy; ^2^ Unit of Molecular Therapies, Department of Experimental Oncology and Molecular Medicine, Fondazione IRCCS Istituto Nazionale dei Tumori, Milan, Italy; ^3^ Department of Theoretical and Applied Sciences, Università degli Studi dell'Insubria, Busto Arsizio, Italy; ^4^ Division of Molecular Oncology and Department of Onco-Hematology, IRCCS Ospedale San Raffaele, Milan, Italy; ^5^ Università Vita-Salute San Raffaele, Milan, Italy; ^6^ Present address: Molecular Biotechnology Center and Department of Molecular Biotechnology and Life Sciences, University of Turin, Turin, Italy

**Keywords:** RNase, Ovarian cancer, Microenvironment, Stress response

## Abstract

As widely recognized, tumor growth entails a close and complex cross-talk among cancer cells and the surrounding tumor microenvironment. We recently described the human *RNASET2* gene as one key player of such microenvironmental cross-talk. Indeed, the protein encoded by this gene is an extracellular RNase which is able to control cancer growth in a non-cell autonomous mode by inducing a sustained recruitment of immune-competent cells belonging to the monocyte/macrophage lineage within a growing tumor mass. Here, we asked whether this oncosuppressor gene is sensitive to stress challenges and whether it can trigger cell-intrinsic processes as well. Indeed, RNASET2 expression levels were consistently found to increase following stress induction. Moreover, changes in RNASET2 expression levels turned out to affect several cancer-related parameters *in vitro* in an ovarian cancer cell line model. Of note, a remarkable rearrangement of the actin cytoskeleton organization, together with changes in cell adhesion and motility, emerged as putative mechanisms by which such cell-autonomous role could occur. Altogether, these biological features allow to put forward the hypothesis that the RNASET2 protein can act as a molecular barrier for limiting the damages and tissue remodeling events occurring during the earlier step of cell transformation.

## INTRODUCTION

The human *RNASET2* gene encodes a highly conserved and secreted ribonuclease which acts as a tumor suppressor in several cancer models [1-6]. In an ovarian cancer model, we recently found that such oncosuppressive role relies on RNASET2-mediated *in vivo* recruitment of cells from the monocyte/macrophage lineage in the tumor mass [5, 6]. Such non-cell autonomous role as a tumor suppressor was also suggested by the observed RNASET2-mediated chemotactic properties toward cells of the monocyte/macrophage lineage [6]. This finding is in keeping with several recent reports which showed a modulation of the innate immune system carried out by other members of the T2 extracellular ribonucleases family, such the *Schistosoma mansoni* Omega-1 protein [7, 8]. A role for human *RNASET2* in establishing a correlation between tumor initiation/progression and modulation of the immune system was also inferred following the recent finding that the oncogenic virus HTLV-1 *tax* gene product drives a strong down-regulation of *RNASET2* gene expression [9]. Furthermore, molecular correlates of *RNASET2*-associated biological responses were recently provided by our group following investigations in an ovarian cancer cell-based xenograft model, where *in vivo* gene expression profiling disclosed a significant *RNASET2*-dependent modulation of expression for gene categories related to immune response functions [6], strongly suggesting a role for RNASET2 as an alarmin-like molecule. The latter represent endogenous danger-signaling molecules that are either passively secreted by necrotic cells or actively secreted by leukocytes or epithelial cells in order to signal the occurrence of tissue-damaging events, such as infection-mediated injury [10, 11]. Taken together, the hypothesis of *RNASET2* as an early determinant of tumorigenesis, coupled to its behaviour as a putative alarmin-like molecule, prompted us to investigate whether this gene could be involved in microenvironmental stress response, possibly acting as a sensor of cellular damage, as recently described for the *Saccharomyces cerevisiae* ortholog of *RNASET2* (Rny1p), which plays an important role in the response to oxidative stress [12].

In the present work, we show indeed that RNASET2 responds to several stress conditions (in particular hypoxia) by being upregulated and actively secreted in the extracellular environment, where it is presumed to carry out its oncosuppressive role [5, 6]. However, a stress-dependent altered dynamics of RNASET2 intracellular isoforms was disclosed as well. The latter findings prompted us to carry out an in depth analysis of the previously underestimated intracellular roles of RNASET2, in order to investigate novel mechanisms through which this mainly extracellular RNase might also operate in a strictly cell-autonomous mode.

Here, we present data indicating that RNASET2-knockdown actually affects several *in vitro* cancer-related parameters that are compatible with a cell-autonomous role for RNASET2 in tumor growth control under stress conditions. Significantly, the observed cell-intrinsic roles of RNASET2 might in part operate through the control of both the cytoskeletal actin assembly (in keeping with the known role of members of the T2 RNase family as actin-binding proteins) [2, 13, 14] and cell motility/migration patterns.

Altogether, our data suggest that, besides the widely recognized non-cell autonomous oncosuppressive role carried out by extracellular RNASET2, a clear cell-autonomous function which might significantly enhance the tumor suppressive activity of this protein is also detected in cancer cells which express this protein endogenously.

By providing evidence that RNASET2 levels are increased in cancer cells under stress conditions and by showing that RNASET2 expression might have profound effects on several cancer-related parameters in the same cells, our data provide a more detailed insight into the cellular bases for the *in vivo* oncosuppressive role played by this protein.

## RESULTS

### *RNASET2* secretion by human ovarian cancer cells is required for *in vivo* tumor suppression

Our previous results in ovarian cancer models strongly suggested a primarily non–cell autonomous role for RNASET2, whose *in vivo* tumor suppressive activity was shown to rely on the modulation of the host immune response [5, 6]. As secretion of RNASET2 seemed to represent a critical step for its biological function, we further validated this hypothesis in our previously established ovarian cancer xenograft model [5]. To this end, the coding region of *RNASET2* was engineered to introduce a KDEL endoplasmic-reticulum retention signal in order to prevent RNASET2 secretion, and the KDEL-modified RNASET2 construct was transfected into the Hey3Met2 human ovarian cancer cell line, which expresses very low levels of endogenous RNASET2 [5].

**Figure 1:**
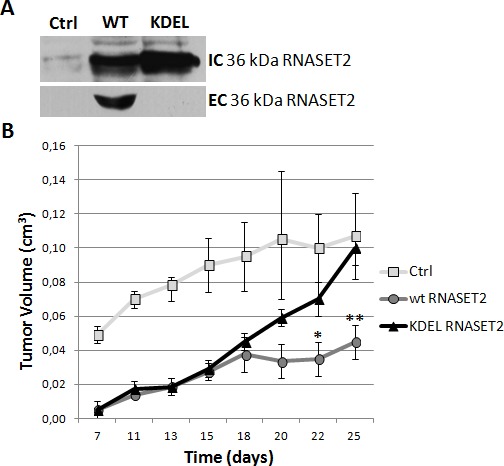
RNASET2 secretion is required for *in vivo* tumor suppression Hey3Met2 cells clones stably transfected with pcDNA3 vector (Ctrl), wild-type (WT) and KDEL-RNASET2-expressing vectors were inoculated in nude mice and tumor growth was evaluated every 2-3 days. A) Western blot analysis on cell extracts and cell culture supernatants from single clones, showing complete inhibition of secretion of KDEL-modified RNASET2 protein. IC: intracellular; EC: extracellular. B) *In vivo* tumor growth kinetics from three clones for each experimental group.

As shown in Figure 1A, the KDEL-modified RNASET2 protein was easily detected in Hey3Met2 cell extracts but not in cell culture supernatants following stable transfection, confirming that it cannot be secreted. Strikingly, unlike wild-type *RNASET2* cDNA-transfected cells, Hey3Met2 cells overexpressing KDEL-modified RNASET2 were not suppressed in their tumorigenic potential, giving rise to large tumors whose size was similar to that observed in control Hey3Met2 cells, which do not express *RNASET2* (Figure 1B).

The apparent lag phase in the emergence of KDEL-modified RNASET2-expressing tumors during the first 15 days after inoculation was possibly due to a secretory pathway overload caused by the accumulation of KDEL-modified RNASET2 in the endoplasmic reticulum, although we cannot formally exclude the possibility that such effect could alternatively be attributed to an intrinsic oncosuppressor role for RNASET2. To rule out that the large tumors grown from KDEL-modified RNASET2 overexpressing cells were the result of a silencing event affecting the transfected transgene, a western blot analysis with an anti-RNASET2 antibody was carried out on excised tumor samples. This assay showed a strong RNASET2 expression in tumors derived from cells transfected with both wild-type and KDEL-modified RNASET2 expression vectors (data not shown). Taken together, these data provide a clear support for RNASET2 secretion as a crucial step for *in vivo* tumor suppression.

### *RNASET2* behaves as a stress-response gene in cultured ovarian cancer cell lines

Since the *Saccharomyces cerevisiae* ortholog of *RNASET2* (Rny1p) has recently been involved in the response to oxidative stress in yeast [12], we hypothesized that *RNASET2* could also behave as a stress-response gene.

**Figure 2:**
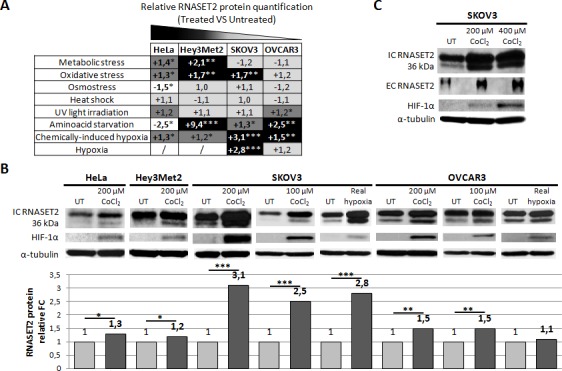
Human RNASET2 protein levels change following stress induction A) Eight different stress-inducing conditions were applied to four different cell lines and Western blot analysis for RNASET2 protein was performed. Relative fold-changes in RNASET2 expression are reported in treated *vs.* untreated cells, with dark and light shadows representing higher or lower levels with respect to untreated cells, respectively. B, C) Western Blot analysis data on total protein extracts (B) and supernatants (C). A significant increase in the levels of the intracellular 36 kDa RNASET2 protein was observed in response to both chemically-induced (CoCl_2_) and real (1% O_2_) hypoxic conditions. HIF-1α protein levels were assessed as an hypoxia stress marker. A concomitant increase of the secreted 36 kDa RNASET2 protein (EC RNASET2) was also observed in SKOV3 cells in response to chemically-induced hypoxia. Triplicate experiments were performed for each experimental condition assessed. Statistical analysis was performed using two-tailed Student's *t*-test. *p<0,05; **p<0,01; ***p<0,001. UT: untreated. IC: intracellular. EC: extracellular. FC: fold-change.

Several stress conditions were therefore applied to a panel of human ovarian cancer cell lines (Hey3Met2, SKOV3 and OVCAR3) and the non-ovarian HeLa cell line and protein levels were subsequently assessed by immunoblotting. Stress conditions included heat shock, aminoacid starvation, UV irradiation, hypoxia, metabolic, oxidative and osmotic stresses. A summary of the results is shown in Figure 2A. Overall, we observed a clear trend for an increase in RNASET2 expression in ovarian cells under several stress conditions. Specifically, RNASET2 protein levels were increased in response to: 1) hypoxia, metabolic stress, oxidative stress and aminoacid starvation in Hey3Met2 cells; 2) hypoxia, oxidative stress and aminoacid starvation in SKOV3 cells, and 3) hypoxia, UV irradiation and aminoacid starvation in OVCAR3 cells. By contrast, either an increase or a decrease in RNASET2 expression was detected in HeLa cells depending on the stress condition applied. Noteworthy, chemically-induced hypoxia represented the only stress condition for which an increase in RNASET2 expression was found for all cell lines tested (Figure 2B).

Despite the observed trend for a general increase in RNASET2 expression in ovarian cancer cells, this response involved different isoforms of the protein, depending on the nature of applied stress. Thus, for aminoacid starvation and metabolic or oxidative stress, an increase in RNASET2 expression was detected for the 27 and 31 kDa intracellular forms [15] (Supplementary Figure 1) whereas hypoxic stress affected mainly the 36 kDa isoform of the protein. Since the latter represent the full-length and secreted form of RNASET2 [15], we validated this result by showing an increase of RNASET2 levels in SKOV3 cell supernatants following hypoxic stress (Figure 2C).

Taken together, these results suggest a general role for the human *RNASET2* as a stress-response gene in human ovarian cancer cells. To corroborate this hypothesis, we carried out a detailed *in silico* analysis of both a 5 kb region immediately upstream of the first exon of the *RNASET2* gene and the whole first intron. Analysis of these genomic regions with the PROMO algorithm (http://alggen.lsi.upc.es/cgi-bin/promo_v3/promo/promoinit.cgi?dirDB=TF_8.3) showed two putative HIF-1 binding sites positioned 466 base pairs (sequence: ACGTGCCGT) and 279 base pairs (sequence: ACGTGCGCG) upstream of the ATG start codon of the gene. Moreover, a further putative HIF-1 binding site (sequence: ACGTGCCTT) was predicted 3443 base pairs downstream of the ATG start codon, within the RNASET2 gene's first intron (data not shown). Although these putative binding sites have not been experimentally validated, their occurrence in the surrounding of the gene's transcript start site suggests a putative role for one or more of these elements in the observed stress-induced upregulation of RNASET2 expression.

Interestingly, the observed increase in RNASET2 secretion in response to hypoxia is in keeping with its putative role as an alarmin. However, since some stress conditions triggered a marked increase also in the intracellular 27 and 31 kDa isoforms, some previously unknown cell-autonomous roles for RNASET2 might also be present. These data, coupled with the high degree of phylogenetic conservation of T2 ribonucleases and the relevant stress-related intracellular roles described for several T2 RNase family members [16], prompted us to investigate whether similar intracellular behaviors can be ascribed to *RNASET2* in mammalian cells.

### RNASET2 affects the *in vitro* growth potential of ovarian cancer cells

Several *in vitro* parameters related to cancer growth were next evaluated, using the previously described RNASET2-silenced OVCAR3 ovarian cancer cell line as experimental model [6].

**Figure 3:**
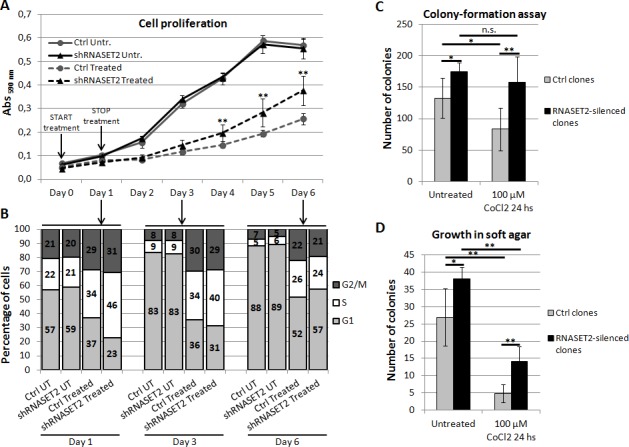
RNASET2 affects several cancer-related parameters in OVCAR3 cells following chemically-induced hypoxia A) A cell proliferation assay was performed in RNASET2-silenced (shRNASET2) and scrambled control OVCAR3 cell clones (Ctrl), following a 24-hours treatment with 100 μM CoCl_2_ (start in Day 0). RNASET2-silenced clones showed no difference in proliferation rate when compared to control clones. By contrast, RNASET2-silenced clones showed a proliferation rate significantly higher than control clones during recovery from chemical hypoxia. Triplicate experiments were performed with four RNASET2-silenced and four ctrl OVCAR3 cell clones. B) A flow cytometry analysis was performed in RNASET2-silenced and scrambled control OVCAR3 cell clones stained with propidium iodide, following a 24-hours treatment with 100 μM CoCl2. C) RNASET2-silenced OVCAR3 cell clones generate a greater number of colonies than control cell clones in colony formation assay. Control clones, but not their RNASET2-silenced counterpart, were significantly affected in their clonogenic capability by CoCl_2_ treatment. D) RNASET2 significantly affects anchorage-independent growth in two-layer soft agar assay, in both normal culture conditions and chemically-induced hypoxia. Triplicate experiments were performed with four RNASET2-silenced and four control OVCAR3 cell clones. Statistical analysis was performed using two-tailed Student's *t*-test. *p<0,05; **p<0,01; n.s.: not significant.

First, we compared the *in vitro* cell proliferation rate of *RNASET2*-silenced (shRNASET2) clones to that of control clones (Ctrl) following a 24-hours treatment with 100 μM CoCl_2_ (Figure 3A). In unstressed cells, *RNASET2*-silenced clones showed no difference in proliferation rate when compared to control clones. However, under hypoxic conditions the proliferation rate of *RNASET2*-silenced clones was significantly higher when compared to controls during recovery from chemical hypoxia.

To investigate the nature of the observed increase in the proliferation rate in *RNASET2*-silenced cells under hypoxic conditions, cell cycle distribution was assessed by flow cytometry following PI staining. As shown in Figure 3B and Supplementary Figure 2A, in the absence of stress we could not detect any significant differences between control and *RNASET2*-silenced OVCAR3 cells in the percentage of cells in G1, S or G2/M phases. This situation was maintained throughout the observation period and is in keeping with the observed overlap in the growth kinetics of these two cell populations. By contrast, at the end of the 24-h exposure to CoCl_2_ a marked increase in the percentage of cells in S phase was observed in both control and *RNASET2*-silenced cells, likely due to a hypoxic stress-mediated block in S phase. Interestingly, at 24h the effect of treatment was more evident in *RNASET2*-silenced cells, which showed a higher percentage of S phase cells (Figure 3B). However, during the five-days recovery period from hypoxic stress, *RNASET2*-silenced cells seemed to resume the cycling pattern typical of unstressed cells more readily than control cells, which probably accounts for their respective growth kinetics.

We then extended our panel of *in vitro* assays to include the ability of *RNASET2*-silenced OVCAR3 cells to form colonies in culture dishes and to grow in an anchorage-independent fashion, both of which resulted significantly increased following hypoxic stress with respect to control clones (Figure 3C-D and Supplementary Figure 2B-C). Noteworthy, these two parameters were increased in *RNASET2*-silenced OVCAR3 cells also in the absence of stress, suggesting that even under optimal culture conditions high *RNASET2* expression levels can substantially affect several cancer-related parameters.

Finally, we tested whether *RNASET2* silencing could affect apoptosis. As shown in Supplementary Figure 3, in the absence of stress *RNASET2-*silenced OVCAR3 cells showed a three-fold decrease in the number of apoptotic cells when compared to control cells, although the fraction of apoptotic cells was very small in both samples. Such minimal apoptotic rate trend was also observed when OVCAR3 cells were treated with CoCl_2_, suggesting that this type of stress was not significantly affecting the apoptotic rate. However, when OVCAR3 cells were challenged with a more intense and non-physiological apoptogenic stimulus (100 nM cis-platinum), the percentage of apoptotic cells was significantly increased in control OVCAR3 cells, whereas *RNASET2*-silenced cells were significantly protected from apoptosis (Supplementary Figure 3).

Taken together, these *in vitro* data strongly suggest that, besides the non cell-autonomous role previously reported *in vivo* [5, 6], the oncosuppressive activity of *RNASET2* gene might be associated with cell-autonomous roles which are apparently exacerbated by hypoxia.

### RNASET2 protein re-localizes to P-bodies following induction of chemical hypoxia

**Figure 4:**
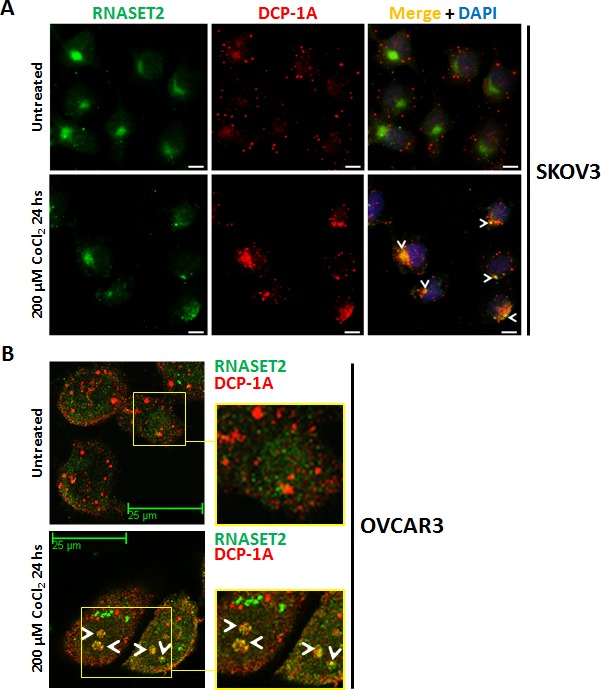
RNASET2 protein re-localizes to P-bodies in response to chemically-induced hypoxia A) SKOV3 cells were seeded on coverslips, treated with 200 μM CoCl_2_ for 24 hours and then processed for double IIF. No co-localization signal between RNASET2 and DCP-1A was detected in untreated SKOV3 cells, while a partial overlap (white arrowheads) was detected following treatment. Scale bar: 10 μm. B) A partial re-localization of RNASET2 protein to P-bodies (white arrowheads) was also detected in OVCAR3 cells following treatment. Confocal microscopy images. Scale bar: 25 μm. DCP-1A: de-capping enzyme 1A (PBs marker).

In a previous work, we have shown that the intracellular RNASET2 protein pool is in part localized into Processing-bodies [17]. We thus re-evaluated these data in the context of the present results. As SKOV3 cells showed the highest increase in RNASET2 expression following stress induction (Figure 2B), we evaluated whether hypoxic stress in this cell line was associated with RNASET2 subcellular localization changes. As shown in Figure 4A, in the absence of stress no co-localization was observed between RNASET2 and the P-bodies marker DCP-1A. However, under hypoxic conditions, the RNASET2 protein showed a more punctuated cell pattern and a partial overlap with the DCP-1A signal, suggesting that a fraction of the RNASET2 intracellular pool is re-directed to P-bodies. The intracellular localization of RNASET2 in response to hypoxic stress was also evaluated in OVCAR3 cells, where a partial re-localization of RNASET2 to P-bodies was observed as well (Figure 4B). Of note, no localization changes for RNASET2 were detected with respect to lysosomes and stress granules under the same stress conditions (data not shown). These data suggest that the RNASET2 stress response role might in part be mediated by engagement of this protein to P-bodies.

### The RNASET2 protein induces a profound cytoskeletal re-organization

In order to assess whether *RNASET2* silencing might trigger other intracellular phenotypic changes, we turned to cytoskeletal changes, on the basis of previous data for the *Schistosoma mansoni* RNASET2 ortholog Omega-1, which promotes a consistent change in mouse dendritic cells morphology as a result of cytoskeletal rearrangements [7]. Similar results were also reported for human colon cancer cells treated with the RNASET2 fungal ortholog ACTIBIND [13]. In both reports, the actin network was apparently targeted by this ribonuclease [7, 13].

**Figure 5:**
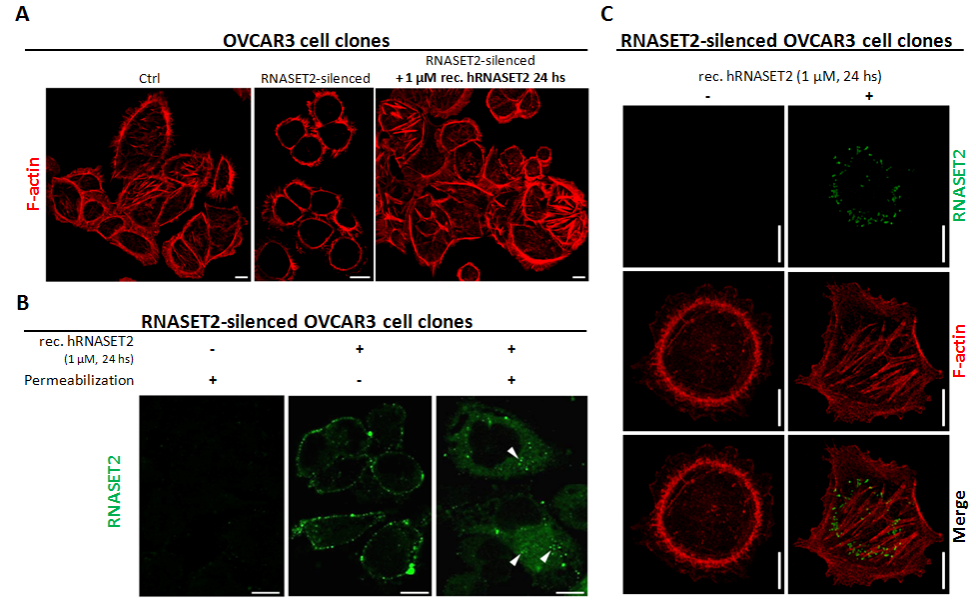
RNASET2 protein affects the structural organization of the actin cytoskeleton and is readily internalized by OVCAR3 cells A) OVCAR3 cell clones were seeded on coverslips and stained with TRITC-conjugated Phalloidin. A complex network of actin filaments is evident in control OVCAR3 cells, while the actin cytoskeleton appears disrupted in RNASET2-silenced cells. After addition of recombinant RNASET2, RNASET2-silenced OVCAR3 cells restored the original cytoskeletal pattern. Scale bar: 10 μm. B, C) RNASET2-silenced OVCAR3 cells were seeded on coverslips, treated with recombinant RNASET2 protein and then processed for IIF. B) In unpermeabilized cells, RNASET2 localizes to the cell surface, while after cell permeabilization a cytoplasmic signal was detected, consistent with the internalization of the protein (white arrowheads). C) Co-staining RNASET2 protein with phalloidin-TRITC showed no evident colocalization signal between the F-actin and the internalized protein. Confocal microscopy images. Scale bar: 10 μm.

We thus investigated the cytoskeletal organization of phalloidin/TRITC-stained control OVCAR3 cells. As shown in Figure 5A, the pattern of actin staining was indicative of a complex network of actin filaments and stress fibers, with several long actin-filament bundles crossing the cell length. Strikingly, such pattern was significantly disrupted in *RNASET2*-silenced OVCAR3 cells, which showed a predominant pattern of peripheral actin filament bundles, indicating a significant rearrangement of actin stress fibers in the absence of RNASET2 (Figure 5A). Since endogenously expressed RNASET2 is effectively secreted by OVCAR3 cells, we wondered whether the observed re-organization of the cytoskeleton could be affected by a trans-acting mechanism mediated by extracellular RNASET2. Indeed, treatment of *RNASET2*-silenced OVCAR3 cells with recombinant RNASET2 readily restored the original cytoskeleton pattern detected in control cells (Figure 5A). By contrast, microtubule organization was not affected by *RNASET2* silencing (Supplementary Figure 4), indicating a specific role for RNASET2 in the actin cytoskeleton.

To further investigate this RNASET2-mediated *trans*-acting process, we evaluated whether the exogenously-provided protein could interact with the OVCAR3 cell surface. Indeed, as shown in Figure 5B, recombinant RNASET2 protein was clearly able to bind the cell surface of *RNASET2*-silenced OVCAR3 cells. Moreover, a cytoplasmic RNASET2 signal was detected following cell permeabilization, indicating RNASET2 internalization by these cells (Figure 5B), although co-staining of recombinant RNASET2 with phalloidin/TRITC did not show co-localization between the actin cytoskeleton and the internalized protein (Figure 5C). Altogether, these data indicate that RNASET2 internalization by OVCAR3 cells is associated with a significant re-organization of the actin cytoskeleton. We also carried out a time-course immunofluorescence assay by evaluating the kinetics of RNASET2 binding/internalization at different times (24, 48 and 72 hours) following addition of recombinant RNASET2 protein to *RNASET*2-silenced OVCAR3 cells. The results further confirmed both the cell surface binding and subsequent internalization of RNASET2 (Supplementary Figure 5). Interestingly, the cell surface signal for RNASET2 was more evident at 24 hours after the addition of the recombinant protein than in later time points. As expected, the opposite trend was observed for the intracellular RNASET2 signal, which increased during time and was therefore consistent with a model of RNASET2 cell surface binding followed by internalization. In keeping with our previous results, no evident co-localization between the internalized RNASET2 protein and the phalloidin-TRITC staining was observed at any time point, thus supporting an indirect effect of the RNASET2 protein on the organization of the actin cytoskeleton.

### Silencing of RNASET2 in OVCAR3 affects cell motility and focal adhesions dynamics

**Figure 6:**
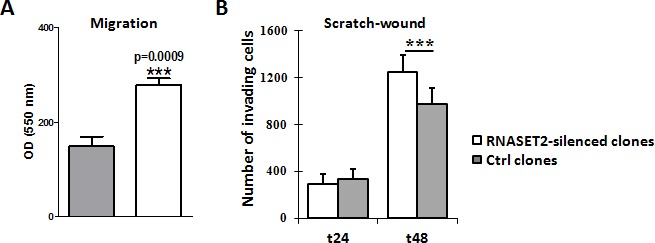
Silencing of RNASET2 in OVCAR3 affects cell motility A) A migration assay performed on pools of control vs. *RNASET2*-silenced OVCAR3 cells showed a significant increase in cell motility in *RNASET2*-silenced cells. B) A scratch test on the same cells also showed an increase in cell-motility in RNASET2-silenced cells when compared to control OVCAR3 cells at 48 hours after wounding. Asterisks indicate statistical significant values analyzed by Student's *t*-test. *** p<0,001.

We next investigated whether the observed RNASET2-mediated changes in actin cytoskeleton might also affect cell migration and invasion *in vitro*. To this end, control and *RNASET2*-silenced OVCAR3 cells were tested by a transmigration assay. Starved cells were seeded onto the transwell chambers and the migratory capability induced upon serum stimulation was monitored after 48 hr. *RNASET2*-silenced OVCAR3 cells showed 2-fold increased migration rate than the corresponding scrambled cells (Figure 6A). A scratch wound assay was also carried out as an independent test for migration, by measuring the area of the wounded surface at different time points. Again, *RNASET2*-silenced OVCAR3 cells turned out to show an increased cell-motility rate when compared to control cells (Figure 6B).

**Figure 7:**
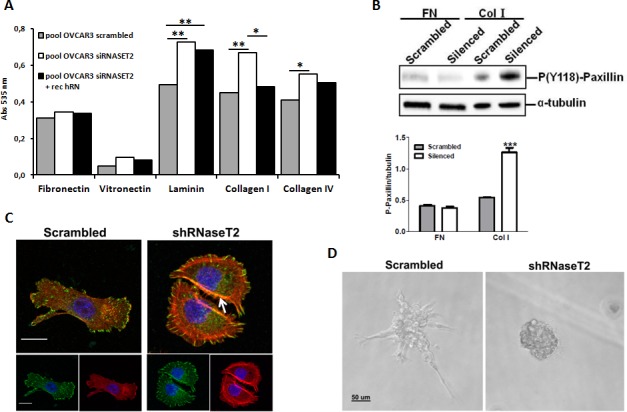
RNASET2 silencing affects both cell adhesion and FA dynamics in OVCAR3 cells A) A colorimetric *in vitro* cell-adhesion assay was performed on five different ECM components. Significant differences in cell-adhesion on laminin, collagen type I and collagen type IV were observed when RNASET2-silenced OVCAR3 cell clones were compared to control clones. Such differences turned out to be weakened when RNASET2-silenced cells were exposed to recombinant RNASET2 protein. Triplicate experiments were performed with four RNASET2-silenced and four control OVCAR3 clones. Statistical analysis was performed using two-tailed Student's t-test. * p<0,05; ** p<0,01. B) Evaluation of phoshoryated paxillin on total cell lysates upon adhesion on fibronectin (FN) or collagen type I (Col I). Upper panel: immunoblotting for the expression of phospho-paxillin. Lower panel: densitometric analysis reporting the levels of phosphorylated paxillin with respect to α-tubulin expression of cells grown on ECM matrices. Statistical analysis of data from triplicate experiments was performed using Student's *t*-test. *** p< 0.001. C) Confocal IIF performed on migrating OVCAR3 cell pools. Starved cells were induced to migrate through a wound upon stimulation with FCS for 24 hr, and then fixed with paraformaldehyde. FAs were stained with anti-phoshphorylated paxillin (green); actin was stained with phalloidin (red). Nuclei were counterstained with DAPI. The white arrow indicates dorsal stress fibers present in RNaseT2-silenced cells. Scal bar: 50 μm. D) Control vs. RNASET2-silenced OVCAR3 cells were grown in reduced growth factor-matrigel 3D culture and pictures were taken with a light microscope.

To evaluate whether the observed effects of RNASET2 on cell morphology and migration could be modulated by a differential activation of extracellular matrix/integrin signaling, we also carried out an *in vitro* cell adhesion assay on control and *RNASET2*-silenced OVCAR3 cells grown on different ECM-derived components. As shown in Figure 7A, we could not detect any significant difference in cell adhesion on fibronectin or vitronectin. However, cell adhesion to laminin and collagens type I and IV was markedly increased in *RNASET2*-silenced OVCAR3 cells. Moreover, recombinant RNASET2 was able to partially restore a reduced adhesion in the latter cells, particularly with respect to collagen I (Figure 7A).

In addition, the adhesion of *RNASET2*-silenced cells on collagen type I was shown to increase the phosphorylation of paxillin (Figure 7B), an ECM/integrin-activated component of focal adhesions (FAs), indicating that the lack of RNASET2 might affect this signaling pathway. Since cell migration is known to be strictly associated with the presence of actin stress fibers in close cooperation with focal adhesions (FAs) [18], we decided to evaluate actin cytoskeleton assembly together with that of FAs in migrating cells. To this purpose, both starved control and *RNASET2*-silenced OVCAR3 cells were stimulated to migrate through a wound upon serum stimulation. A confocal IF analysis was then performed to analyze actin stress fibers and FAs. Upon exposure to the migratory stimuli, *RNASET2*-silenced OVCAR3 cells showed a strong staining of dorsal stress fibers (Figure 7C) which were not detectable in control OVCAR3 cells. Staining with anti-phosphorylated paxillin evidenced fully mature FAs, compatible with increased actin stress fiber-mediated tension and active migration in *RNASET2*-silenced cells. By contrast, control OVCAR3 cells showed a more mesenchymal-like morphology with smaller and less elongated FAs, together with the capability to extend to form lamellipodia and filopodia (Figure 7C).

One of the characteristics of mesenchymal-like cells is to produce proteases able to degrade the ECM and to invade. To further investigate the molecular and functional differences between control and *RNASET2*-silenced cells, we performed reduced growth factor-matrigel-embedded 3D culture to induce cell aggregation and formation of spheroids, with the aim to mimic an invasion process similar to that occurring *in vivo* during cancer cell invasion. Both control and *RNASET2*-silenced OVCAR3 cells were able to form tridimensional structures, but the latter formed compact spheroids with numerous filopodia, while control OVCAR3 formed a stellate phenotype characterized by filopodia and chains of cells invading through the surrounding matrigel (Figure 7D), which is one of the characteristics of mesenchymal-like cells. These results indicate that the lack of RNASET2 affects the structure of both actin cytoskeleton and FAs, thus contributing to a change from a mesenchymal to an amoeboid cell morphology, which in turn might led to a different behavior during migration and invasion. These data argue for the hypothesis that RNASET2 contributes to mechanisms of signaling associated to integrin-based adhesion.

## DISCUSSION

A wide range of environmental stress conditions represent early steps in the pathogenesis of several diseases, including cancer. Indeed, cells typically respond to stress challenges by the activation of several pathways, whose molecular features have been thoroughly investigated over the last decades [19]. Quite recently, within the conceptual context of stress response, special attention has been devoted to the activation of ribonucleolytic enzymes in both prokaryotes and higher organisms. For instance, a consistent stress response in yeast cells under a wide range of stress conditions has been shown to entail the production of tRNA and rRNA fragments by means of stress-activated ribonucleases [20]. In this context, the yeast ortholog of *RNASET2* (Rny1) is activated following oxidative stress by release from the cell vacuole [12] and the presence of this RNase in the cytosol is the pre-requisite for its biological functions. These early stress-response processes seem to be remarkably conserved, as a similar mechanism is also at work in mammalian cells [20]. In this case, the RNase angiogenin, which is released from the nuclear compartment or dissociates from an inhibitor in the cytoplasm, plays a major role [21]. The biologically relevant issue here is that RNase release is one of the first steps following stress challenge and can therefore be considered as an early marker of cellular damage.

In this context, the similarity in Rny1p and RNASET2 actions (the latter complementing Rny1p absence in yeast cells) [12] also extends to the observation that some of the roles played by these enzymes are independent of their ribonuclease activity. Thus, catalytically-impaired Rny1p still elicits a reduction of yeast cells viability and activates apoptosis when released from the vacuole [12]. This is in agreement with our recent data on a mouse model of ovarian carcinoma, where catalytically-inactivated RNASET2 was still able to control ovarian carcinogenesis [22]. Of note, similar results were also obtained by other investigators with the fungal ortolog of RNASET2, ACTIBIND [13]. Altogether, these findings suggest that, besides angiogenin, RNASET2 could also be implicated in stress response. However, since a formal proof of this hypothesis was still lacking, we provide here experimental evidence that this is indeed the case.

The investigation of the processes where RNASET2 is involved following stress challenges that we report here highlighs the pleiotropic functions of this phylogenetically conserved protein, which are typical of multitasking/moonlighting proteins [23]. Indeed, several of these multitasking roles are reported for RNASET2 in this work.

To begin with, we have investigated the effect of a variety of stress conditions on OVCAR3, Hey3Met2 and SKOV3 ovarian cancer cells and on the non-ovarian HeLa cell line, respectively. All cell lines responded to chemically-induced hypoxia by increasing RNASET2 expression, whereas other stress conditions elicited a differential response. As expected, the extracellular 36 kDa RNASET2 isoform was readily induced and secreted, but also the intracellular localized 27 and 21 kDa isoforms turned out to be upregulated upon stress. The latter finding clearly pointed to the occurrence of specific RNASET2-mediated cell-autonomous processes, besides those associated with the secreted 36 kDa isoform. Exploitation of the *RNASET2*-silenced OVCAR3 model system, already known to be endowed with a tumorigenic propensity *in vivo* [6], was crucial for an in-depth analysis of the intracellular dynamics concerning this protein in unstressed and stressed conditions.

One of the first functions we ascribed to this multitasking protein following stress challenge concerns the cell cycle. Indeed, although hypoxic stress affected cell cycle dynamics by increasing the percentage of OVCAR3 cells in S-phase independently of RNASET2 expression status, once the hypoxic challenge was over the presence of RNASET2 appeared to slow down the proliferative recovery of control cells, while *RNASET2*-silenced cells were apparently able to readily resume cell cycle progression. Hypoxia and hypoxia mimetics are generally reported to alter cell cycle progression in cultured cells, although the nature of the response is rather unpredictable and seems to depend on the specific cell type. Thus, while a minority of cell lines seems to be unaffected [24-28], G1/S arrest is most commonly observed. Recently, a decrease in G1 phase and an increase in S and G2/M phase following CoCl_2_ treatment have been reported [29, 30]. Our results appear to agree with these last reports. This is basically in keeping with what has been found in the yeast system, where overexpression of Rny1 and also of the ortolog *RNASET2* gene in mutant yeast cells affects both cell growth and viability and sensitizes cells to oxidative stress [12].

Another important process where Rny1/RNASET2 play a role in yeast cells, which underscore an independent role for these proteins, is induction of apoptosis, which does not require any ribonuclease catalytic activity [12], suggesting that induction of apoptosis is a distinct function of these RNases independent of the production of cleaved RNAs. We report here similar findings with RNASET2 and additionally show that the observed RNASET2-mediated response is apparently related to the strength of the stress challenge. Indeed, with chemically induced hypoxia (CoCl_2_ at 100 μM) the observed apoptotic response was rather weak, whereas treatment with platinum elicited a strong apoptotic response. It is thus tempting to speculate that under low stress doses, which result in a moderate increase of RNASET2 levels, this RNase likely contributes with angiogenin to cleave tRNAs and rRNAs resulting in cell cycling changes that somehow contribute to neutralize the stress stimulus [20]. However, under more challenging stress conditions, a much increased RNASET2 level might mediate a marked switch in cell fate, resulting in cell death. Both Rny1 and *RNASET2* seem therefore endorsed with a cell damage response function associated with cell survival following stress challenges.

Additionally, the latter finding unveiled a further action of RNASET2, namely in the context of P-bodies, where this protein was found to relocalize upon stress challenges. Since P-bodies represent intracellular sites where RNA turnover takes place and a complex mRNPs dynamics in these intracellular structures underlies cell response following stress challenges [31], our data further suggest that the *in vitro* response observed in ovarian cancer cells under hypoxic conditions might involve RNASET2-mediated processing of transcripts within these subcellular structures.

Being RNASET2 a clear multitasking protein, we reckoned that other intracellular processes could be affected by this protein. Indeed, close inspection of cell morphology revealed a rather profound RNASET2-mediated change in the cytoskeleton, since the actin structural network was consistently rearranged by *RNASET2* silencing in OVCAR3 cells. Strikingly, the observed dynamics was basically in keeping with the previously reported *Schistosoma mansoni* Omega-1-mediated cytoskeleton changes [7]. It is worth noting that the observed lack of co-localization between RNASET2 and the actin cytoskeleton was quite unexpected. Indeed, the protein encoded by the RNASET2 ortholog from the mold *Aspergillus niger* has been given the name ACTIBIND, due to its ability to bind actin [13]. Moreover, the same actin-binding role was reported for human RNASET2 by the same research group [2]. However, the reported interactions between actin and T2 RNase proteins were assessed only by ligand blotting or in solution interaction assays, which represent poorly physiologic conditions. Our immunofluorescence assays, while reporting a strong effect of RNASET2 on the actin cytoskeleton, challenges the notion of a direct RNASET2-actin interaction in the cell cytoplasm and suggest the occurrence of an indirect effect of the RNASET2 protein on the organization of the actin cytoskeleton. Although the molecular mechanisms by which RNASET2 indirectly affects the cell cytoskeleton has not been investigated here, we reckon that internalized RNASET2 might interact (possibly via its C-terminal region) with intracellular protein partners that can in turn affect the assembly or dynamics of the cytoskeleton, as described below.

We also provide evidence that RNASET2 is involved in the stabilization of actin stress fibers, as *RNASET2*-silenced cells apparently lose their mesenchymal shape. Moreover, 3D culture in Matrigel allowed us to clearly define that RNASET2 contributes to stress fiber assembly and to the establishment of the mesenchymal mode of invasion. Of note, accumulating evidences indicate that 3D cultures are more representative of ovarian cancer disease conditions than classical bidimentional assays [32], since they may provide a useful *in vitro* approach to analyze morphological changes with respect to proliferative and invasive capability of tumor cells respectively. We have here demonstrated that the lack of RNASET2 was associated with both an increase in anchorage-independent growth in agar (in line with our previous demonstration that *RNASET2* expression is inversely correlated to ovarian cancer cell growth in *in vivo* mouse models) [5, 6] and a different pattern of cell migration and invasion in Matrigel, which was never assessed before. Of note, although our confocal IF assays did not assess a direct binding of RNASET2 to actin stress fibers, distinct actin assembly mechanisms of stress fiber subtypes have been hypothesized involving myosin II as well as actin binding proteins such as mDia1 and Arp2/3 protein complex [33]. Presently, we cannot exclude an association between RNASET2 and these cytoskeletal-associated molecules, therefore the clarification of these mechanisms deserve further and more specific investigations.

Finally, an intriguing result that emerged in this work (which in our opinion deserves further investigations) is the observed binding of RNASET2 to the cell surface. A putative receptor for the human RNASET2 protein has not been reported in the literature so far. Moreover, no reports for cell surface receptors have been published for any protein belonging to the T2 RNase family, apart for Omega-1 T2 RNase from the helmint parasite *Schistosoma mansoni*, which was recently shown to bind the mannose receptor in human dendritic cells [34]. Some very preliminary data from our group support the putative interaction of RNASET2 with a cell surface G-protein coupled-like receptor (GPCR), since the chemotactic activity of RNASET2 on human monocytes was significantly impaired when these cells were pre-treated with pertussis toxin [6]. Based on these data, efforts are ongoing in our laboratory to isolate such putative human RNASET2 receptor in OVCAR3 cells.

Altogether, these observations suggest a highly pleiotropic role for RNASET2 in tumor suppression, whereby several independent cellular parameters related to cancer growth are affected by changes in the expression levels on this protein. Whereas some of these biological responses are readily apparent following stress induction (such as the increased secretion of RNASET2 to orchestrate an alarmin response or the observed changes in cell proliferation rate) and might therefore represent a defense strategy against cancer-inducing stress conditions such as hypoxia, others are apparently at work even in the absence of stress.

Overall, the present data open a new avenue for understanding the pathobiology of solid tumors and in particular of ovarian cancer. In this regard, transformation of the ovarian surface or the tubal epithelia, the tissues from which the ovarian carcinoma is thought to originate [35], seem to be favored by an inflammatory microenvironment which together with hypoxia causes genotoxic damage in neighboring cells [36]. It is tempting to speculate that RNASET2 can act as a molecular barrier for limiting the damages and tissue remodeling occurring during the earlier step of transformation [37].

## METHODS

### Cell lines and clones

HeLa, Hey3Met2 (4), OVCAR3 and SKOV3 cells were cultured in DMEM-F12 medium with 10% FBS and 1% L-glutamine. Hey3Met2 cells were stably transfected with a pcDNA3-based expression vector encoding human *RNASET2*, as previously described (4). This expression vector was modified by standard PCR mutagenesis to derive an *RNASET2* coding sequence with a C-terminal KDEL motif for endoplasmic reticulum retention. The resulting KDEL-RNASET2 expression vector was stably transfected in Hey3Met2 cells as well.

OVCAR3 cell clones stably-transfected with empty pSicoR vector, control scrambled shRNAs and two RNASET2-targeting shRNAs were already described (5).

### Antibodies and fluorescent dyes

Primary antibodies: rabbit polyclonal anti-hRNASET2 (Dabio); mouse monoclonal anti-HIF-1α (Abcam); mouse monoclonal anti-DCP1A, mouse monoclonal anti-calnexin and mouse monoclonal anti-YBX1 (Abnova); mouse monoclonal anti-RCK/p54 and mouse monoclonal anti-LAMP2 (Santa Cruz Biotechnology); rabbit polyclonal anti-Pp38 and rabbit polyclonal anti-p38 (Cell Signaling Technology); anti-phosphopaxillin (Y118, Invitrogen). Goat anti-mouse IgG HRP-conjugated and anti-rabbit IgG HRP-conjugated (PIERCE); mouse monoclonal anti-α tubulin, goat anti-mouse IgG TRITC-conjugated and goat anti-rabbit IgG FITC-conjugated (Sigma-Aldrich).

### *In vivo* xenograft assays

Male nude (5 animals/group) mice were challenged subcutaneously (s.c.) in the left flank with 5x 10^6^ Hey3Met2 cells (parental, empty vector-transfected or expressing wild-type or KDEL-modified RNASET2) in 0.15 ml of medium and 0,15 ml Matrigel® (Sigma). Animals were monitored twice a week for weight and tumor growth (measuring three perpendicular diameters), and sacrificed when the mean tumor volume of control mice reached a dimension of ≥100 mm^3^. Tumors samples were snap-frozen and kept at −80°C for protein extraction. Animals were maintained in a pathogen-free facility and treated in accordance with European Union guidelines under the approval of the ethical committee of Ospedale San Raffaele (Institutional Animal Care and Use Committee protocol 418).

### Stress-inducing procedures

Stress induction on cell lines was applied using sodium (meta)arsenite, clotrimazole, D-sorbitol, Dulbecco's Modified Eagle's Medium High Glucose without L-methionine, L-cysteine and L-glutamine and Cobalt(II) chloride (all from Sigma-Aldrich). Metabolic stress was induced by treatment with 500 μM NaAsO_2_ for 45 minutes, followed by a 120-minutes recovery. Oxidative stress was induced by 1-hour incubation with 20 μM clotrimazole diluted in serum-free medium. Osmotic shock was induced with 1 M D-sorbitol for 30 minutes in either serum-free or complete medium. Heat shock was carried out by 20-minutes incubation at 42°C, followed by a 2-hours recovery period. UV-light exposure was performed with UVB lamps at 312 nm for 1 minute, followed by a 30-minutes recovery. Aminoacid starvation was induced by 48-hours incubation with DMEM with 4.5 g/l glucose and NaHCO_3_, without Methionine, Cysteine and Glutamine. Chemically-induced hypoxia was induced by incubation with 100-400 μM CoCl_2_ for either 12 or 24 hours. Real hypoxic conditions were obtained in culture chambers with 1% O_2_ concentration for 24 hours.

### Western blot analysis

Cultured cells were mechanically scraped in PBS + 5mM EDTA and resuspended in lysis buffer (0.5% Igepal, 0.5% Triton X-100 in PBS + 5mM EDTA) supplemented with a protease inhibitors cocktail. For SDS-PAGE analysis, 30-70 micrograms of intracellular lysates were loaded for each lane. Immunoblot analysis was performed using standard procedures and detection was performed with a chemiluminescent substrate (Super-signal *West Dura*, Thermo Scientific).

### *In vitro* assays

For cell proliferation assays, 10^3^ cells/well were plated in 96-wells culture plates and treated for the induction of chemical hypoxia. After 24-hours the medium was replaced and absorbance values at 595 nm were measured daily over a 7-days period using the CellTiter® 96 non-radiactive kit (Promega).

For colony-formation assays, 2×10^2^ cells/well were plated in six-wells culture plates and treated with 100 μm CoCl_2_ for 24 hours. Following a 10-14 days incubation, clones were stained with 1% methylene blue/50% ethanol and manually counted.

Anchorage-independent growth was assessed by a two-layer soft-agar test. Following a 24-hours treatment with CoCl_2_, 100 cells were resuspended in the over-layer (0.3% Difco Agar Noble (Becton-Dickinson) in DMEM/F12 supplemented with 20% FBS) and then plated over the under-layer (0.6% Difco Agar Noble in DMEM/F12 supplemented with 20% FBS) in 24-wells culture plates. After 16 days, colonies were stained with 0,1% Crystal Violet and manually counted.

For scratch-wound assay, 5×10^5^ cells per well were seeded in 6-wells plates and grown to confluence. A scratch was then performed on the monolayer with a pipette tip. Images of the same fields were taken 12, 24 and 48 hours after the scratch and analyzed using the TScratch software (ETH Zürich). The effective number of migrated cells was manually counted.

For migration assay, starved cells (1,5×10^5^) were seeded on Transwell® inserts (8μm, Corning Life Science) and migration was induced by adding FCS in the lower chamber. Migrating cell were counted after 48 hr.

Cell adhesion to extracellular matrix (ECM) components was assessed using the Millicoat ECM® screening kit (Millipore). Briefly, 5×10^4^ cells per well were seeded in 96-wells culture plates coated with ECM components. After 1-hour incubation, wells were washed and cells were stained with 0.2% crystal violet solution, solubilized and the absorbance values were determined at 540-570 nm on a microplate reader.

For the evaluation of phosphorylated paxillin, starved cell were harvested by trypsin and seeded (3×10^5^) on fibronectin or collagen type I plates (Gibco, Life Technology). After 30 min, all cells were collected and lysed with 1x SDS-PAGE sample buffer and analyzed by western blotting.

For 3D culture, cells (1×10^3^) were suspended in medium growth factor-reduced Matrigel® (BD Biosciences) and then seeded directly onto uncoated 48-well culture plates. Plates were first incubated for 30′ at 37°C and then complete medium was added. Morphological changes in spheres shape was monitored up to 10 days using an inverted microscope. Images were acquired with ACT-1 software (Nikon).

### Cell cycle analysis

Following CoCl_2_ treatment, 5×10^5^ cells were fixed in 0,5 ml of ice-cold 100% ethanol, centrifuged, washed with PBS, resuspended in the dying solution (50 μg/ml Propidium Iodide, 20 μg/ml RNase in 1X PBS) and analyzed with FACSCalibur flow cytometer (Becton-Dickinson). Data were processed using CellQuest software (Becton-Dickinson).

### Indirect immunofluorescence (IIF) assays

Cells were grown on coverslips for 24 hours and treated or directly processed for indirect immunofluorescence. Briefly, cells were fixed in 3% paraformaldehyde and permeabilized using Triton X-100; alternatively, they were fixed in 100% methanol. After blocking (10% goat-serum in PBS), incubations with antibodies were performed in 3% goat-serum in PBS. Cell nuclei were counterstained with DAPI and coverslips were mounted on microscope slides. Images were taken using a Leica TCS SP8 X confocal laser scanning microscope and acquired in 512×512 pixel scan format in a Z stack series (step size 0.5 μm). The data were analyzed using the software Leica LAS AF rel. 3.3

### Statistical analysis

Either two-tailed Student's *t*-test or two-way ANOVA were used to analyze data from both *in vivo* and *in vitro* assays for statistical significance, assuming p<0,05 as a threshold value to discard the null hypothesis.

## SUPPLEMENTARY MATERIAL FIGURES


