# Supplementary material for: Pleiotropic modes of action in tumor cells of RNASET2, an evolutionary highly conserved extracellular RNase
Source: Oncotarget. 2015 Mar 8;6(10):7851–65. doi: 10.18632/oncotarget.3490 (PMC4480721; doi:10.18632/oncotarget.3490)
Supplement: Supplementary file 1 [file oncotarget-06-7851-s001.pdf]

Pleiotropic modes of action in tumor cells of RNASET2, an evolutionary highly conserved extracellular RNase

Supplementary Material

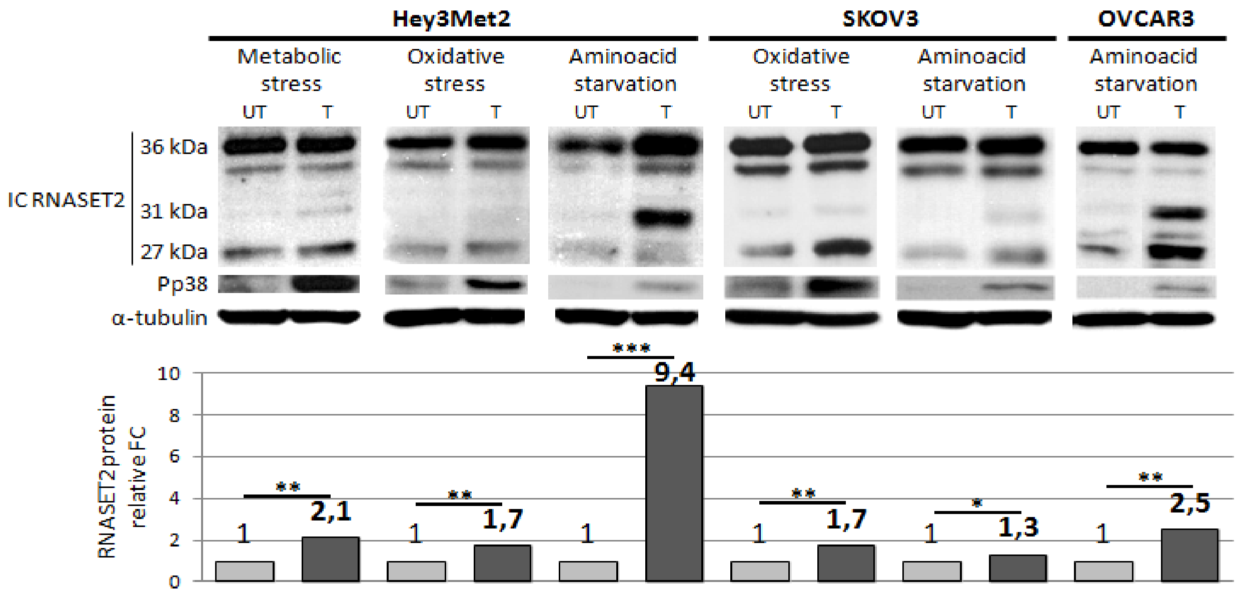

Supplementary figure 1: Specific intracellular isoforms of RNASET2 protein are induced in response to stress. Immunoblot analysis was performed on total protein extracts obtained from both treated and control cells, following stress induction. Under metabolic or oxidative stress and aminoacid starvation, an increase in RNASET2 was detected for the 27 and 31 kDa intracellular forms of RNASET2. Phosphorylated-p38 protein was assessed as a general stress marker. Triplicate experiments were performed for each treatment. The graph below shows the fold-increase values for each treatment. Statistical analysis was performed using two-tailed Student's *t*-test. *p<0,05; **p<0,01; ***p<0,001. UT: untreated. T: treated. IC: intracellular. FC: fold-change.

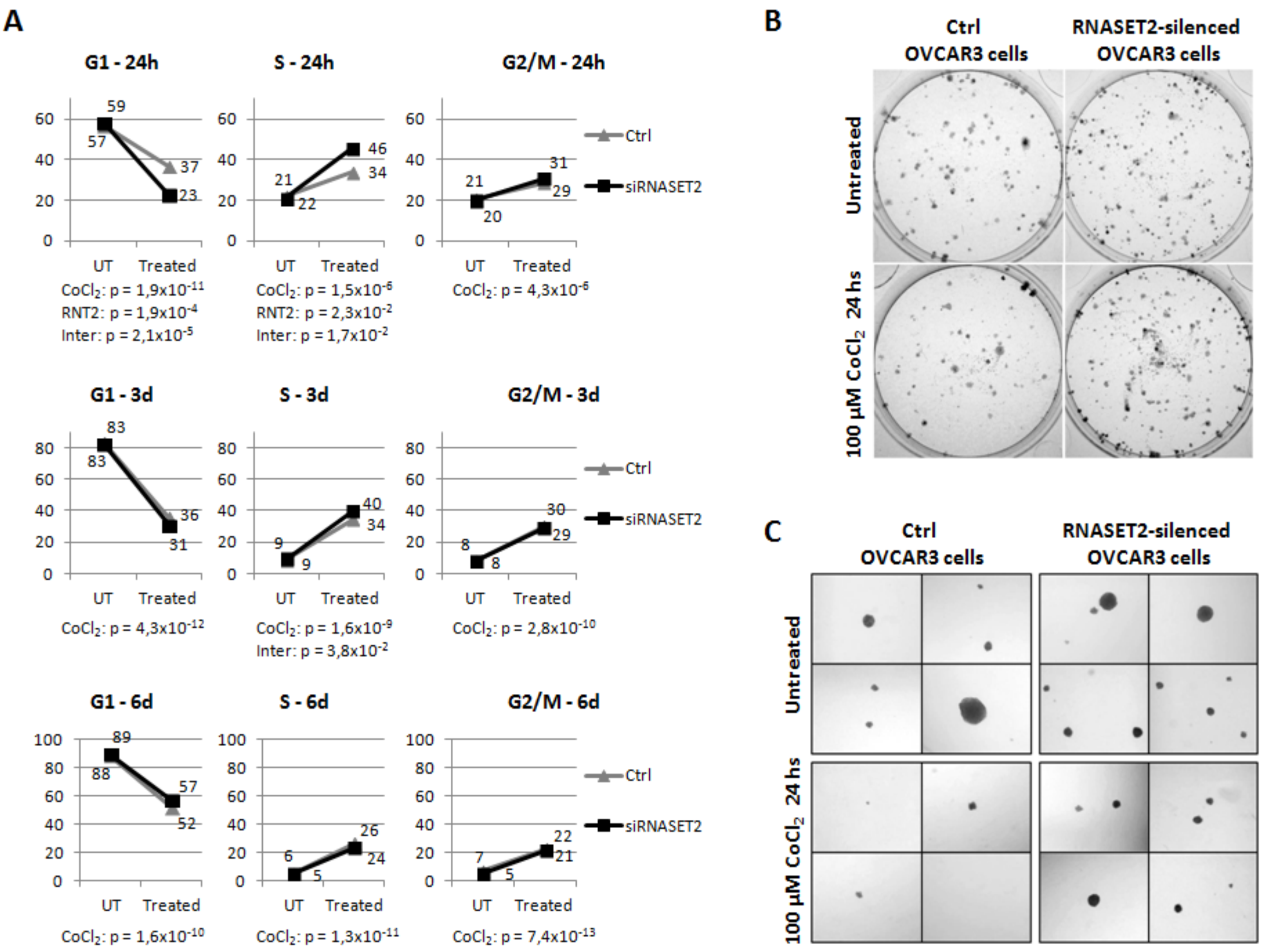

Supplementary figure 2: Cell cycle phase transitions and clonogenic activity/anchorage-independent growth are affected by RNASET2 upon chemical hypoxia. A) Statistically significant differences were induced by both treatment and RNASET2 expression in G1 and S phases. Moreover, the interaction between the two factors (treatment and RNASET2) was also significant 24 hours after the $CoCl_2$ treatment, suggesting that RNASET2 affects the sensitivity to the early S-phase blockade induced by $CoCl_2$ treatment. Experiment was performed with four RNASET2-silenced and four ctrl OVCAR3 clones. Statistical analysis was performed using two-way ANOVA. **B)** Representative plates from clonogenic assays showing that control OVCAR3 clones, but not their RNASET2-silenced counterpart, were significantly affected in their clonogenic capability by $CoCl_2$ treatment. **C)** RNASET2 significantly affects anchorage-independent growth in two-layer soft agar assay, in both normal culture conditions and chemically-induced hypoxia.

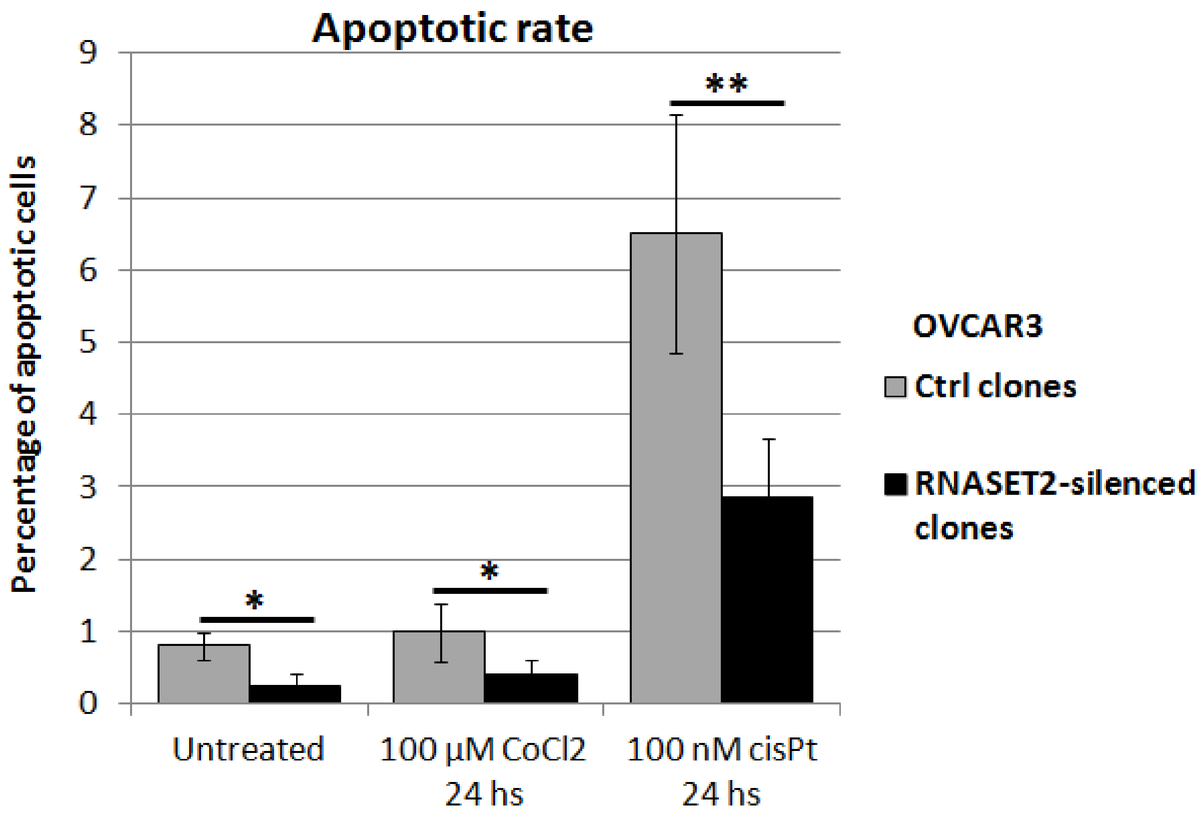

Supplementary figure 3: RNASET2 expression affects cell-death rate in response to pro-apoptotic stimuli. Flow cytometry analysis was performed in RNASET2-silenced OVCAR3 cell clones with respect to control clones stained with propidium iodide, following a 24-hours treatment with either cobalt chloride or cis-platinum. In all of the three experimental conditions, RNASET2-silenced clones showed a percentage of apoptotic cells significantly lower than control clones. Two experiments were performed with four RNASET2-silenced and four ctrl OVCAR3 cell clones. Statistical analysis was performed using two-tailed Student's *t*-test. *p<0,05; **p<0,01.

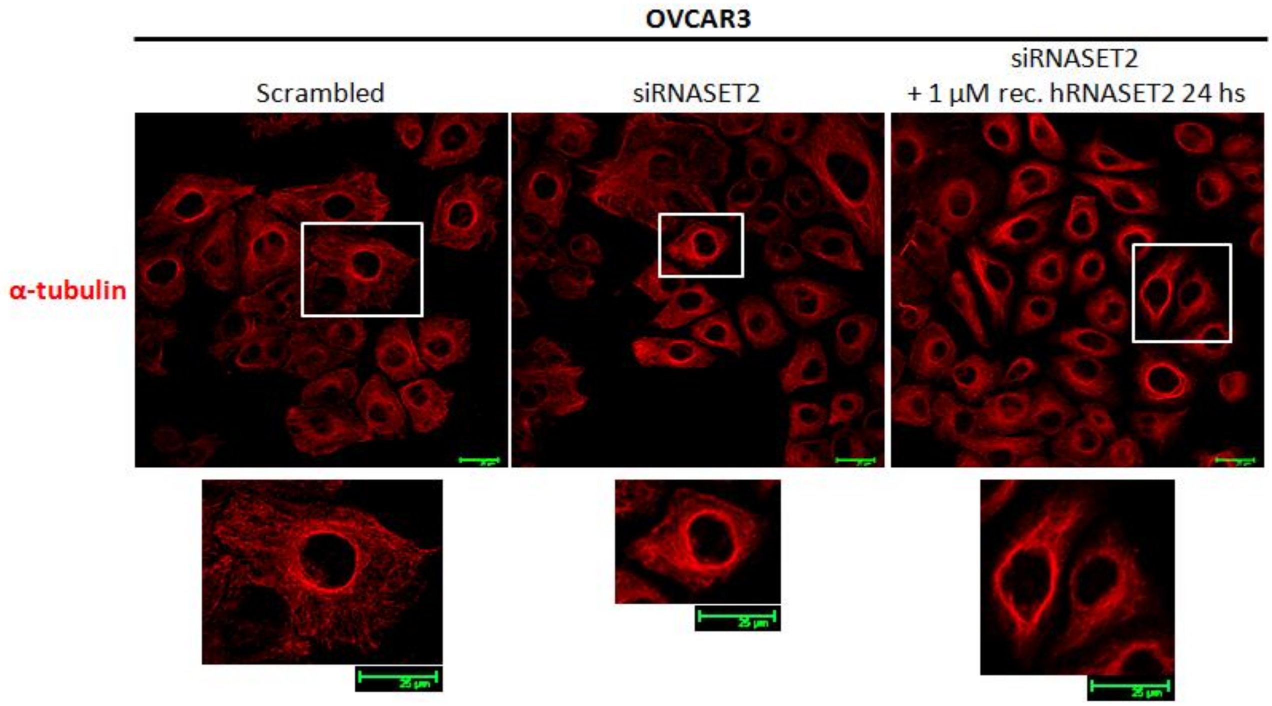

Supplementary figure 4: Microtubule organization is not affected by RNASET2. IIF assay for α-tubulin was performed on control vs. *RNASET2*-silenced OVCAR3 cells. No differences in the organization of microtubules were induced by RNASET2. Confocal microscopy images. Scale bar: 25 µm.

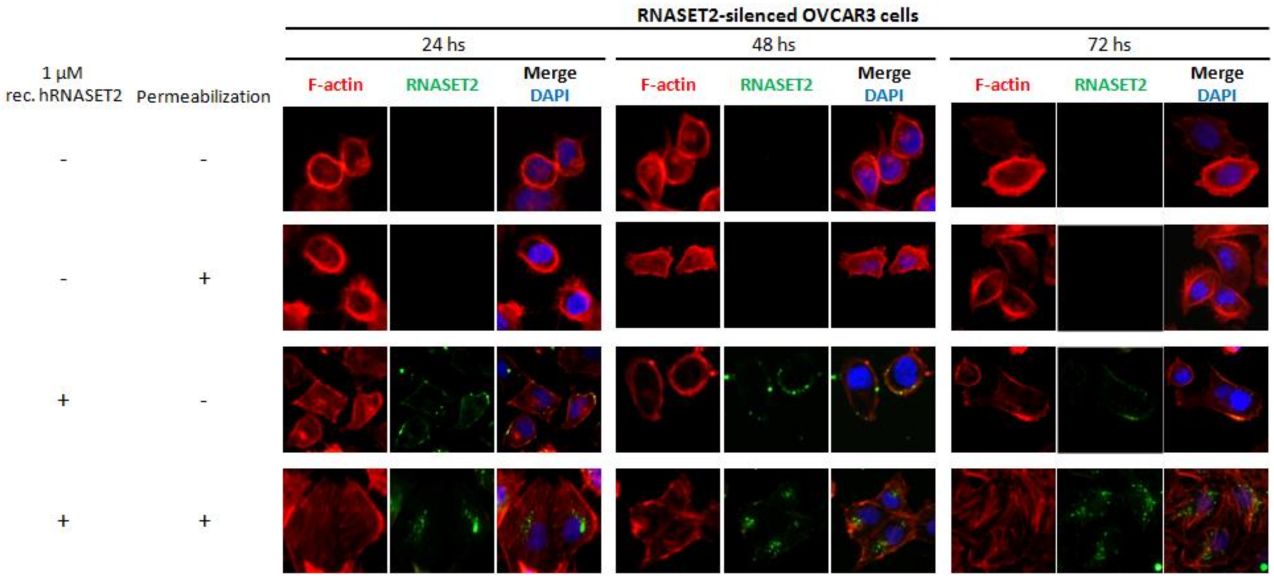

Supplementary figure 5: RNASET2 binding/internalization kinetics in RNASET2-silenced OVCAR3 cells. RNASET2-silenced OVCAR3 cells were seeded on coverslips, treated or not with 1 µM recombinant RNASET2 protein (24-, 48-, 72-h incubation) and stained with both TRITC-conjugated Phalloidin (red) and an anti-RNASET2 antibody (green). In untreated cells (first and second rows of the panel) no RNASET2 signal was detected. In RNASET2-treated unpermeabilized cells (third row) RNASET2 localizes to the cell surface. In RNASET2-treated permeabilized cells (fourth row) the internalization of the protein is evident with a more intense and punctuated signal in later time points. The actin cytoskeleton rearrangement was observed following treatment with the recombinant protein but no evident co-localization of the RNASET2 signal with F-actin was detected in any conditions assessed. Fluorescence microscopy images.
